# Integrated Analysis of *Salmonella* Infantis in Chicken Meat: Epidemiological Surveillance, Antibiotic Resistance, and Potential Bioactive Control Agents

**DOI:** 10.3390/pathogens14111178

**Published:** 2025-11-18

**Authors:** Yasin Tekin, Hatice Yazgan, Tulin Guven Gokmen, Nuri Gungor, Nur Sima Uprak

**Affiliations:** 1 General Directory of Meat and Milk Board, Adana Meat Directory, Adana 01330, Türkiye; yasin.tekin@esk.gov.tr (Y.T.); vetnurigungor@gmail.com (N.G.); 2 Department of Food Hygiene and Technology, Faculty of Ceyhan Veterinary Medicine, Cukurova University, Adana 01330, Türkiye; 3 Department of Microbiology, Faculty of Ceyhan Veterinary, Cukurova University, Adana 01330, Türkiye; tulinguven01@hotmail.com (T.G.G.); nuprak@cu.edu.tr (N.S.U.)

**Keywords:** *Salmonella* Infantis, antibiotic resistance, carvacrol, alpha terpineol, eugenol

## Abstract

*Salmonella* species isolated from chicken meat pose an increasing threat to public health. According to ECDC data, salmonellosis cases have shown a significant upward trend in many European countries between 2019 and 2023, almost reaching pre-pandemic levels. EFSA reported 77,486 confirmed human cases in the EU in 2023. This corresponds to a notification rate of 18 cases per 100,000 people, compared to 15.4 cases per 100,000 in 2022. This study evaluated the prevalence of *Salmonella* spp., antimicrobial resistance (AMR) profiles, and the effectiveness of natural biological preservatives in raw chicken meat obtained from retail outlets in Southeast Turkey. Among 100 samples analyzed according to ISO 6579-1:2017, suspicious colonies were detected after selective enrichment in XLD and n = 3 isolates were confirmed to be *Salmonella enterica* subsp. *enterica* serovar Infantis by real-time PCR. Disk diffusion tests performed in accordance with EUCAST showed that all isolates were resistant to beta-lactam, tetracycline, trimethoprim, sulfonomid and aminoglycoside groups. All isolates were classified as multidrug-resistant. PCR detected *blaTEM-1* (all isolates), *aphA1-IAB* (all isolates), *aadA1* (two isolates), and *sul1* (all isolates), while *tetA*/*tetB* genes were not detected. Among the natural compounds tested, carvacrol showed the strongest antimicrobial activity (MIC 1.56 µL/mL; MBC 3.125–6.25 µL/mL; inhibition zones 32–35 mm). Eugenol showed moderate effects with higher MIC/MBC values (3.125–6.25 µL/mL/12.25 µL/mL), while α-terpineol was effective only at higher concentrations. These findings are consistent with the global increase in *Salmonella* Infantis and AMR, supporting carvacrol followed by eugenol and α-terpineol as promising natural alternatives for controlling MDR *Salmonella* spp. in food safety applications.

## 1. Introduction

*Salmonella* spp. are among the most prevalent Gram-negative, foodborne zoonotic bacteria and represent a leading cause of gastrointestinal infections in humans worldwide [1]. The most common route of human infection is the consumption of contaminated food, particularly chicken and eggs, while pork and other meat products are less frequently implicated. Contact with infected animals or contaminated environments can also result in transmission [2,3].

It is reported that, between 200 million and 1 billion *Salmonella* spp. infection cases occur annually, resulting in 93 million gastroenteritis cases and approximately 155,000 deaths. Foodborne transmission accounts for about 85% of these cases [4,5]. *Salmonella* spp. cause salmonellosis, a disease characterized by fever, diarrhea, vomiting, and abdominal pain, typically appearing 6–48 h after the ingestion of contaminated food. Approximately 5% of patients experience additional symptoms, and the illness generally lasts between four and seven days [4,6]. More than 60,000 cases of nontyphoidal salmonellosis were reported in the European Union (EU) in 2021 [7]. Fewer than 1% of the 100 million people estimated to contract *Salmonella* Enterocolitis worldwide each year develop invasive nontyphoidal salmonellosis [8,9]. About 1.4 million instances of salmonellosis are recorded in the US annually, with foodborne illness accounting for the great majority of these cases.

The serotypes *Salmonella* Typhimurium (*S.* Typhimurium) and *Salmonella* Enteritidis (*S.* Enteritidis) notably account for over half of all illnesses [10]. *Salmonella* Dublin has also been related to outbreaks connected to ground beef in addition to these serotypes [11,12]. The *S.* Typhimurium and *Salmonella* Derby serotypes are the most frequently detected in raw pork slices [13]. Nevertheless, another investigation has shown that raw ground pork and raw pork slices could potentially include additional serotypes, such as *S.* Enteritidis, *Salmonella* Infantis (*S.* Infantis), and *Salmonella* Newport (*S.* Newport) [14]. Furthermore, *S.* Infantis has long been a prevalent isolate from poultry in Europe and other countries [9,15,16]. In recent years, it has become increasingly prevalent in broiler chicken populations in many countries, becoming the fourth most common serovar of human salmonellosis in the EU. According to 2022 data, 96% of food animal *S*. Infantis isolates originated from broiler chickens, and this serovar accounted for 50% of all *Salmonella* spp. isolates from broilers [17]. The most prevalent *Salmonella* serotype in Türkiye from 2012 to 2016 was *Salmonella* Enteritidis (57.3–74.1%), followed by Typhimurium (3.0–8.5%), Infantis (4.0–6.7%), Paratyphi B (3.8–3.2%), and Kentucky (3.8–2.7%) [18].

Additionally, salmonellosis also causes significant financial losses in terms of public health and the economy. According to research, the yearly cost is approximately USD 4.1 billion in the United States [19], EUR 3 billion in the EU [20], and AUD 811 million in Australia [21]. This emphasizes the critical importance of adhering to food safety protocols to ensure the proper handling, preparation, storage, and distribution of food appropriately.

One of the most critical public health concerns associated with non-typhoidal *Salmonella* (NTS) is the increasing prevalence of antibiotic-resistant strains. The widespread and often uncontrolled use of antibiotics in human and veterinary medicine, as well as in agriculture and aquaculture, is considered one of the main reasons for the emergence of antibiotic-resistant bacteria [22]. This uncontrolled use facilitates the evolution and dissemination of resistance genes, posing a serious global health threat. This pathogen has developed resistance over time to several critical antibiotic classes, including beta-lactams, aminoglycosides, tetracyclines, and sulfonamides, which are frequently prescribed to treat serious infections. The most frequently detected resistance genes to beta-lactam antibiotics are *blaTEM-1* (ampicillin resistance) and *blaTEM*, *blaSHV*, and *blaCTX-M* (third-generation cephalosporin resistance). For aminoglycoside resistance, *aadA1* and *aphA1-IAB* are the most common genes; for sulfonamide resistance, *sul1* and *sul2*; for tetracycline resistance, *tetA* and *tetB*; and for chloramphenicol resistance, *cat1*, *cat2*, and *floR*. For azithromycin resistance, *mph(A)*, *ramAp*, *erm(B)*, and *erm (42)* have been reported [23,24,25,26].

Antibiotic resistance genes (ARGs) have been found in *Salmonella* spp. strains isolated primarily from meat and meat products, as well as in MDR *Salmonella* spp. Through the food chain, their presence has become a serious threat to human health, particularly with the emergence of multidrug-resistant (MDR) *Salmonella* spp. strains. Investigating the prevalence of *Salmonella* spp. in retail meats is essential for ensuring food safety and preventing economic losses, even though the spread of MDR remains challenging to control [27].

There is growing concern about the negative impacts of chemical preservatives used in the food chain to control these microorganisms harboring resistance genes on food safety and human health. Increasing public awareness of these risks has significantly increased interest in natural antimicrobial alternatives in the food industry [28]. In this context, plant extracts and essential oils with medicinal and antibacterial properties stand out not only for their preservative capacity but also for their potential to preserve the nutritional quality and sensory properties of foods, becoming the focus of current research on sustainable preservation strategies.

Bioactive compounds isolated from plants can be used as natural preservatives against foodborne zoonotic pathogens in various fields, including medicine, veterinary medicine, agriculture, aquaculture, and animal foods such as poultry and red meat. Carvacrol is a natural aromatic bioactive compound found in different plants, such as *Oregano *(*Origanum vulgare* L.) and *Marjoram *(*Origanum majorana* L.), which belong to the *Lamiaceae* family. Carvacrol is a phenolic monoterpene characterized by a strong odor [29]. It has been used in traditional medicine for centuries due to its antimicrobial, antifungal, antioxidant, anticancer, and anti-inflammatory properties. It also has potential applications in the food industry as a natural food preservative against pathogens, as it is classified as generally recognized as safe (GRAS) by the FDA and the European Commission [30,31]. Eugenol, a phenolic aromatic molecule mostly derived from clove oil (*Syzygium aromaticum*), exhibits various biological properties, including antifungal, antibacterial, and anticancer effects [32]. Furthermore, α-terpineol, a volatile monoterpene alcohol, has been investigated for its antibacterial, anticonvulsant, anticancer, insecticidal, anti-inflammatory, and antinociceptive properties, in addition to its potential to extend the shelf life of meat and meat products [33,34,35,36]. α-terpineol is the main component of the essential oils of several aromatic plant species, such as *Origanum vulgare* L. and *Ocimum canum* Sims, which are widely used for medicinal purposes. α-terpineol can also be isolated from various sources, such as cajeput oil (*Melaleuca cajuputi/Melaleuca leucadendra*), pine oil (*Pinus* spp.), and petitgrain oil (*Citrus aurantium* var. *amara*) [37]. Plant materials are typically processed using hydrodistillation, supercritical CO_2_ extraction, or ethanol-based solvent extraction methods. Quantification of the volatile fractions is commonly performed by GC-FID or GC-MS, and compound concentrations are determined in mg/g or % (*w*/*w*) using appropriate internal standards and calibration curves [38].

The present study aimed to comprehensively assess the prevalence, antibiotic resistance profiles, and genomic characteristics of *Salmonella* spp. strains isolated from chicken meat samples obtained from various retail sources. In addition, the antimicrobial effects of selected natural bioactive compounds (carvacrol, eugenol, and α-terpineol) were evaluated in this study for their potential applications in *Salmonella* spp. control within the meat industry.

## 2. Materials and Methods

### 2.1. Salmonella spp. Isolation and Identification

#### 2.1.1. Sample Collection and Isolation

In this study, a total of 100 raw chicken meat samples were collected under aseptic conditions from 25 retail stores located in the central districts of Adana province (Çukurova, Seyhan, Yüreğir, and Sarıçam). Samples representing four product groups, skin-on wings, skin-on breasts, skin-on thighs, and skin-on drumsticks (25 packages each), were obtained with a minimum quantity of at least 500 g. All samples were transported to the laboratory under cold chain conditions on the day of purchase. Conventional *Salmonella* spp. isolation was performed using the standard microbiological procedures described by the International Organization for Standardization (ISO 6579-1:2017) [39], with minor modifications applied according to the method reported by Osivand et al. [23]. For the pre-enrichment process, 25 g of the sample was aseptically weighed directly and placed in sterile filtered stomacher bags (Fisher Scientific, Nepean, ON, Canada), and 225 mL of a Ringer solution was added. For each sample, both surface and internal portions of the meat were included to ensure representative microbiological assessment. Afterwards, the suspension, which was homogenized for 3 min with a stomacher (Masticator, IUL device, Barcelona, Spain), was incubated at 37 °C for 24 h. Pre-enriched inoculum (100 µL) was cultured in 10 mL of Rappaport Vassiliadis Broth (RVB; Merck, Germany) and Tetrathionat Broth (TB). RVB was incubated for 16–18 h at 41.5 °C, while TB was incubated for 24 h at 37 °C. Cultures were taken from MSRV and TB media with a loop and inoculated onto Xylose-Lysine-Deoxycolate (XLD) agar and incubated for 24 h at 37 °C. On XLD agar, fuchsia-colored formations and H_2_S-producing black colonies were evaluated as suspected *Salmonella* spp. isolates.

#### 2.1.2. *Salmonella* spp. Identification

##### DNA Extraction and Real-Time PCR Assay

Genomic DNA was extracted from pure colonies of *Salmonella* spp. grown on Mueller–Hinton Agar using the DNeasy Blood and Tissue Kit with the QIAcube system (Qiagen, Hilden, Germany) and stored at −20 °C. For each sample, a 9 μL PCR master mix was prepared, consisting of 5 μL of 2× qPCR Mix, 3 μL of *Salmonella* spp. Oligo Mix, and 1 μL of inhibition/reaction control. Subsequently, 5 μL of DNA obtained after extraction was added to this mixture. Amplification was performed using a Roche LightCycler 480 real-time PCR system (Roche Diagnostics GmbH, Mannheim, Germany). The cycling procedure included an initial denaturation at 95 °C for 5 min, followed by 12 cycles of denaturation at 95 °C for 1 s and annealing at 67–56 °C for 1 s (touchdown stage). This was followed by 30 cycles consisting of denaturation at 95 °C for 10 s and annealing at 63 °C for 30 s (amplification stage).

### 2.2. Antimicrobial Susceptibility Test

Antimicrobial susceptibility testing was performed using the Kirby–Bauer disk diffusion method in accordance with the European Committee on Antimicrobial Susceptibility Testing (EUCAST). The following antibiotic disks (Bioanalyse Medical, Ankara, Türkiye) were used; Ampicillin (AM, 10 µg), amoxycillin–clavulanic acid (AMC, 10 µg), ceftriaxone (CRO, 30 µg), ceftazidime (CAZ, 30 µg), aztreonam (ATM, 30 µg), cefotaxime (CTX, 30 µg), gentamicin (CN, 10 µg), amikacin (AK, 30 µg), ciprofloxacin (CIP, 5 µg), levofloxacin (LEV, 5 µg), enrofloxacin (ENR, 5 µg), trimethoprim-sulfamethoxazole (TS, 1.25–23.75 µg), tetracycline (T, 30 µg), chloramphenicol (C, 30 µg), streptomycin (STR, 10 µg), and kanamycin (KAN, 30 µg) were used. *Salmonella* spp. isolates incubated at 37 °C for 24 h were standardized to a cell density of 0.5 McFarland. Mueller–Hinton Broth (MHB, Oxoid, CM0405, Basingstoke, UK) was used as the medium. A sterilized cotton swab was dipped into the suspension, and the bacteria were spread evenly over surface of a Muller–Hinton agar plate. Antibiotic disks were then placed on the Muller–Hinton agar (MHA) plates and incubated overnight at 37 °C. Zone diameters were evaluated according to EUCAST [40].

### 2.3. Detection of Resistance Genes by PCR

Resistance genes were detected by PCR using specific primers (Sentebiolab, Bilkent, Ankara, Turkey) and FirePol^®^ Master Mix (Solis Biodyne, Tartu, Estonia), as described in previous studies (Table 1).

### 2.4. The Antimicrobial Effects of Selected Natural Bioactive Compounds (Carvacrol, Eugenol, and α-Terpineol) on Salmonella Isolates

#### 2.4.1. Minimum Inhibitory (MIC) and Bactericidal Concentration (MBC) Assay

Clinical and Laboratory Standards Institute (2008) [51] methods were used to determine the MIC and MBC values of bioactive compounds, including carvacrol, eugenol, and α-terpineol, against three identified *Salmonella* spp. isolates. Test microorganisms incubated at 37 °C for 24 h were standardized to 0.5 MacFarland turbidity. Mueller–Hinton Broth (MHB, Oxoid, CM0405, Basingstoke, UK) was used as the medium. Briefly, 1 mL of the bioactive substance from a 50 mg/mL stock solution was added to the first tube of each series and serially diluted with sterile Muller–Hinton Broth (MHB, Merck, Darmstadt, Germany). The final concentrations of the bioactive compounds were 12.5, 6.25, 3.125, 1.56, 0.78, 0.39, and 0.19 mg/mL. The inoculum suspension (1 mL) of each bacterial isolate was then added to each tube containing bioactive compounds and MHB. Each tube was evaluated for bacterial growth and compared to the control. As a positive control, a tube containing MHB and bacterial suspension without bioactive compounds was used. The tube containing bioactive compounds and bacterial suspension was also used as a negative control. The tubes were then incubated at 37 °C for 24 h. After 24 h, the MIC value was determined by evaluating the turbidity of the growth in the tubes, and 100 µL from each tube was inoculated on MHA Petri plates and incubated overnight at 37 °C. The lowest concentration of the compound at which no growth was observed after one night was determined to be the MBC value.

#### 2.4.2. Agar Well Diffusion Method

In vitro antimicrobial effects of three *Salmonella* spp. isolates were determined using the well diffusion method [52]. Wells with a diameter of 4 mm were utilized as Mueller–Hinton Agar (MHA, Merck, Germany) plates using a sterile cylinder in accordance with the well diffusion method standards. *Salmonella* spp. isolates incubated overnight on Mueller–Hinton agar were suspended in 2 mL of physiological serum, and McFarland turbidity values were standardized to 0.5. Sterile cotton swabs were dipped into the suspensions, impregnating the swabs with bacteria, and excess liquid was then drained from the edge of the tube. The suspension-impregnated swab was spread to cover the surface of the Mueller–Hinton medium. For each bacterial isolate, 100 µL of from stock solution of each food grade bioactive compounds at concentration of 1 g/mL [Carvacrol W224502 (Sigma-Aldrich, Steinheim, Germany), eugenol W246719 (Sigma-Aldrich, Steinheim, Germany), α-terpineol W304522, (Sigma-Aldrich, Steinheim, Germany)] was added to the wells, and the plates were incubated at 37 °C for 24 h. As a control group, only distilled water and MHA were added to the wells of Petri dishes inoculated with each bacterial strain on their plates. All tests were performed in triplicate for each sample. The results were expressed in millimeters (mm) as the arithmetic mean of the inhibition zone diameters around each well.

### 2.5. Statistical Analysis

The obtained data were expressed as mean ± standard deviation (SD). Statistical analyses were conducted using SPSS software version 18.0 (SPSS Inc., Chicago, IL, USA). One-way analysis of variance (ANOVA) and Duncan’s multiple range test were applied to determine significant differences among groups, and correlation variations were also evaluated where applicable. Statistical significance was considered at *p* < 0.05.

## 3. Results

### 3.1. Salmonella spp. Identification

*Salmonella* spp. are considered one of the most prevalent foodborne zoonotic pathogens worldwide and pose a serious public health problem. Despite the success of effective control and eradication programs in developed countries, consumption of contaminated poultry meat and other animal products remains an important source of *Salmonella* spp. infections in humans [53]. In this study, the prevalence of *Salmonella* spp. in raw chicken meat marketed in the Adana province of Türkiye was investigated. A total of 100 conventional raw chicken meat samples, including 25 wings, 25 breasts, 25 drumsticks, and 25 thighs, were collected from 25 different sales points in the Adana province and analyzed for *Salmonella* spp. contamination. The results demonstrated that bacterial growth was detected in all groups inoculated for selective enrichment. Following selective enrichment, suspected *Salmonella* spp. colonies were observed on xylose lysine deoxycholate (XLD) agar after 24 h of incubation at 37 °C. H_2_S-producing black colonies on XLD agar were evaluated as suspect *Salmonella* spp. isolates.

**Figure 1:**
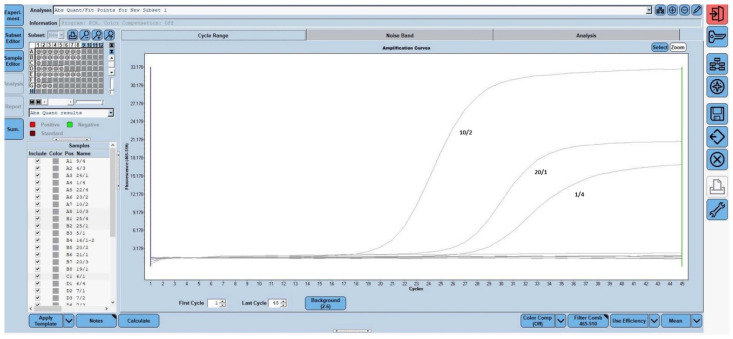
*Salmonella* spp. identification by Real-time PCR method.

Presumptive *Salmonella* spp. isolates obtained from wing, thigh, breast, and drumstick samples were analyzed using the Bio-Speedy *Salmonella* spp. Real-Time PCR Detection Kit (Bioeksen, Istanbul, Türkiye), following the manufacturer’s instructions and employing the Touchdown-PCR protocol. Based on the PCR results, 2 isolates isolated from thighs and 1 isolate isolated from drumstick were confirmed as *Salmonella* spp. The real-time PCR amplification curves of the confirmed positive strains are presented in Figure 1. The colonies confirmed via real-time PCR were further analyzed at the Department of Microbiology, Faculty of Veterinary Medicine, Ankara University, for species-level identification. The results revealed that the isolates were identified as *Salmonella* Infantis (Table 2).

### 3.2. Determination of Antimicrobial Susceptibility

Three isolates of *Salmonella* Infantis were studied for their antibiotic resistance profiles using both phenotypic and molecular methods. All isolates exhibited resistance to ampicillin, amoxicillin–clavulanic acid, tetracycline, trimethoprim–sulfamethoxazole, streptomycin, and kanamycin, as indicated via zone diameter measurements. In contrast, all isolates exhibited susceptibility to aztreonam, cefotaxime, ceftazidime, ciprofloxacin, gentamicin, amikacin, levofloxacin, enrofloxacin, and chloramphenicol (Table 3). Two isolates were resistant to ceftriaxone, and isolate S1 had a zone diameter that was borderline suspicious for ESBL and was also resistant to amoxicillin-clavulanic acid. The detection of resistance to more than one antibiotic class in all isolates suggests that all three isolates can be classified as multidrug-resistant (MDR), posing a potential threat to both food safety and public health (Table 3).

#### Determination of Antibiotic Resistance Genes

**Figure 2:**
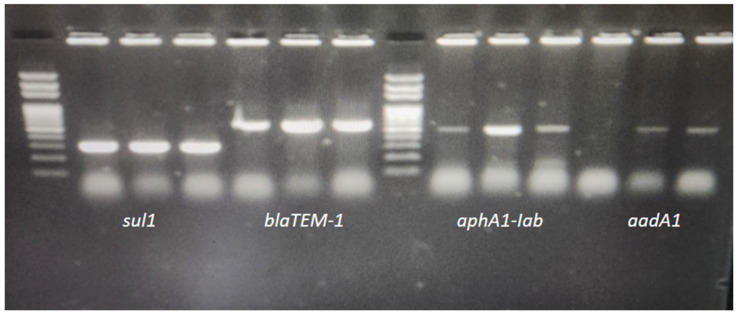
Agarose gel image of resistance genes of *Salmonella* Infantis isolates.

Antibiotic resistance genes were screened in all three isolates detected in this study, and the presence of the *blaTEM-1* gene was detected in three isolates resistant to ampicillin and amoxicillin-clavulanic acid. All isolates in our study showed resistance to streptomycin and kanamycin via disk diffusion, and PCR detection targeting both the *aadA1* and *aphA1-IAB* genes indicated that aminoglycoside-modifying enzymes contribute to this resistance. The *aphA1-IAB* gene was detected in all three isolates, and the *aadA1* gene was detected in two (S2 and S3) (Figure 2).

The phenotype that is resistant to trimethoprim-sulfamethoxazole (TMZ) was confirmed at the molecular level via the detection of the *sul1* gene in all isolates tested in the current study. But, for tetracycline, despite resistance being indicated in the antibiogram, the *tetA* and *tetB* genes were not found.

### 3.3. The Antimicrobial Effects of Carvacrol, Eugenol, and α-Terpineol on Salmonella Isolates

#### 3.3.1. Minimum Inhibitory (MIC) and Bactericidal Concentration (MBC) Assay Values

The current study also evaluated the antimicrobial effects of natural bioactive compounds—including eugenol, carvacrol, and α-terpineol, which may serve as potential alternatives to synthetic preservatives in controlling foodborne pathogens—against three *Salmonella* Infantis isolates obtained from chicken meat. The antimicrobial activities of carvacrol, eugenol, and α-terpineol against *S*. Infantis isolates were evaluated based on their minimum inhibitory concentration (MIC) and minimum bactericidal concentration (MBC) values. Among the bioactive compounds tested on three different *S*. Infantis isolates (S1, S2, and S3), the lowest MIC and MBC values were observed in the carvacrol-treated samples (Table 4).

The MIC value of carvacrol was 1.56 µL/mL for all isolates, while MBC values varied depending on the isolate. These findings indicate that carvacrol exhibits higher antimicrobial activity against *S*. Infantis isolates. The minimum inhibitory concentration (MIC) of carvacrol was determined as 1.56 µL/mL for all isolates, whereas the minimum bactericidal concentration (MBC) values varied among the isolates. S2 and S3 isolates exhibited MBC values of 6.25 µL/mL and 3.125 µL/mL, respectively.

In this study, eugenol showed effective antimicrobial activity despite exhibiting higher MIC and MBC values compared to carvacrol. The MIC of eugenol was determined to be in the range of 3.125–6.25 µL/mL for all isolates, and the MBC value was 12.5 µL/mL (Table 4).

Comparing the α-terpineol MIC and MIC values to the other two compounds in the current work, it was found that this bioactive chemical exhibited the highest activity at high concentrations (Table 4).

#### 3.3.2. Results of Agar Well Diffusion Method 

**Figure 3:**
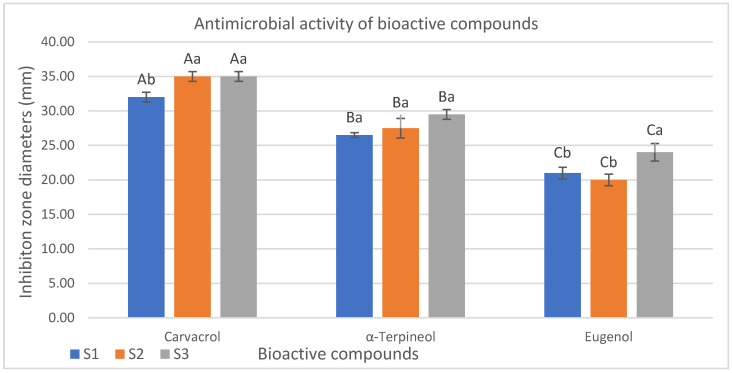
Inhibition zone diameters of carvacrol, alpha-terpineol and eugenol determined by the agar well diffusion method. Values represent mean ± SD. There is a significant difference (*p* < 0.05) between the groups (a,b) and bacteria (A–C) for the values indicated by different letters in the same row and column.

The antimicrobial effects of carvacrol, α-terpineol, and eugenol on *Salmonella* Infantis isolates were assessed based on inhibition zone diameters determined via the agar well diffusion method. Carvacrol demonstrated the strongest antimicrobial activity among the tested compounds, with inhibition zones ranging from 32.00 to 35.00 mm (*p* < 0.05). These findings confirm the potent activity of carvacrol against *Salmonella* spp. strains. The inhibition zone diameters obtained via the agar well diffusion method were evaluated as indicators of antimicrobial activity. Among the isolates tested, S3 was determined to be the most susceptible isolate to α-terpineol with an inhibition zone of 29.50 mm. In contrast, isolates S2 and S1 exhibited slightly lower inhibition zones of 27.50 mm and 26.50 mm, respectively. Although α-terpineol exhibited lower antimicrobial activity compared to carvacrol, statistically significant antibacterial effects were observed against the tested isolates. The lowest inhibition zone diameters were observed in isolates treated with eugenol ranging from 20.00 to 24.00 mm with S3 being the most sensitive isolate (24.00 mm) (Figure 3).

## 4. Discussion

### 4.1. Salmonella Infantis

*Salmonella* Infantis is a serotype that exhibits multidrug resistance (MDR) patterns and is gaining increasing importance among *Salmonella* spp. serotypes originating from poultry meat. When examined epidemiologically, there are significant differences in the prevalence of *Salmonella* spp. in poultry meat between countries. In this study, *Salmonella* Infantis was isolated from 4% of drumstick samples and 8% of thigh samples. In a similar study conducted in Japan, *S*. Infantis was found in 72.2% of ground chicken samples and 84% of boiler samples in Ecuador [54]. In addition, studies conducted in Turkey have shown that the prevalence of this serotype has increased significantly recently [55], suggesting that this serotype may be a source of concern for both poultry production and public health. The prevalence of *S*. Typhimurium and *S*. Enteritidis serovars was also evaluated in our study; however, neither serovar could be detected. Some studies in the literature have investigated the presence of these serovars in edible internal organ samples, but their isolation was not reported, which is consistent with our findings [56,57,58]. In a similar study on 150 chicken meat samples, *Salmonella* spp. was found in 42.66% of the samples, indicating a higher prevalence rate [59]. Babacan and Karadeniz detected *Salmonella* spp. contamination in 35% of packaged chicken meat samples using both the ISO 6579 standard [39] and the Mini Vidas Easy SLM (Biomerieux, Marcy-l’Étoile, France) systems [60]. In Tokyo, *Salmonella* species were reported to be present in 143 of 240 ground chicken samples [61]. Similarly, the prevalence of *Salmonella* spp. is also low. It was reported in 48.3% of 60 chicken meat samples from Surabaya, Indonesia [62]. These findings are consistent with the reports published by the European Food Safety Authority (EFSA) and the European Centre for Disease Prevention and Control (ECDC). According to the One Health Zoonoses Report (2018), the four most common *Salmonella* spp. serotypes isolated from human salmonellosis cases are *S*. Infantis, *S*. Typhimurium, *S*. Enteritidis, and the monophasic variant of *S*. Typhimurium [63]. More recent data presented in the 2022 report indicate that *Salmonella* spp. have been isolated throughout the European Union. Strains isolated throughout the European Union originate from chickens and related food products. The predominant serotypes are *S*. Infantis, *S*. Enteritidis, *S*. Typhimurium, monophasic *S*. Typhimurium, and *S*. Derby [7]. Our findings, which also identified *S*. Infantis as the predominant serotype in chicken meat samples, are consistent with this global upward trend and further emphasize its increasing epidemiological importance.

### 4.2. Determination of Antimicrobial Susceptibility and Antibiotic Resistance Genes

There is a global trend toward increasing ampicillin resistance, particularly among *Enterobacteriaceae* isolates. A study detected high ampicillin resistance in *S*. Infantis isolates [64]. The *blaTEM-1* gene is one of the most prevalent β-lactamase genes and confers resistance to β-lactam antibiotics such as amoxicillin and ampicillin [65]. It forms the evolutionary basis of the extended-spectrum β-lactamase (ESBL) family, and ESBL enzymes originate from these β-lactamases [66]. The presence of *blaTEM-1* in *Salmonella* spp. isolates explains the development of resistance, particularly with respect to first-line antibiotic agents. Furthermore, plasmid carriage of the *blaTEM-1* gene facilitates horizontal gene transfer between species, increasing the spread of resistant *Enterobacteriaceae* within the community [67]. In many countries, the prevalence of *blaTEM-1* in foodborne *Salmonella* spp. strains has been reported to range from 20% to 70% [68,69]. However, in a study examining 135 *S*. Infantis isolates from Japan, the *blaTEM-1* gene was detected in 0.74% [16]. *Salmonella* Infantis strains isolated from poultry meat in Türkiye have been reported to contain additional TEM family enzymes, including *blaTEM-70*, *blaTEM-148*, and *blaTEM-198*. A single nucleotide mutation distinguishes these genes from *blaTEM-1* [70].

In general, *S*. Infantis isolates in this study were found to be susceptible to third-generation cephalosporins, including ceftazidime and cefotaxime. However, *S*. Infantis 1 and 2 isolates showed resistance to cefotaxime (Table 3). Isolate S1 had an inhibition zone of 23 mm, and inhibition zones below 25 mm raised suspicion of ESBL resistance. However, resistance to amoxicillin/clavulanic acid eliminated this suspicion. However, three isolates were screened for the *blaTEM*, *blaSHV*, *blaOXA*, and *blaCTX-M* genes, and none were detectably positive.

Kanamycin and streptomycin resistance in *Salmonella* spp. isolates is clinically important, especially in invasive infections where treatment options are limited. Resistance to these aminoglycosides can often be transmitted via plasmid-borne genes such as *aadA* and *aphA* and can spread to other bacteria. Furthermore, the presence of resistant strains in the food chain poses a serious risk to public health, increasing the importance of antibiotic stewardship strategies. Our findings documented streptomycin and kanamycin resistance in *S*. Infantis isolates at the phenotypic and genetic levels. Multidrug-resistant (MDR) clones resistant to nalidixic acid, streptomycin, sulfamethoxazole, and tetracycline (NaSSuT) have been widely reported in the European poultry industry. Resistant *Salmonella* strains have been documented in many countries, including Hungary, Poland, Austria, Germany, and Switzerland [71,72]. Similarly, Rahmani et al. observed streptomycin resistance in all *S*. Infantis isolates obtained from poultry farms in Iran [73]. Numerous studies have reported a high prevalence of the *aadA1* gene in *S*. Enterica serotypes isolated from poultry [73,74]. The *aadA1* gene was found in 77.14% of *Salmonella* spp. isolates obtained from cloacal swabs and environmental samples at a chicken farm in China, and in Ghana, it was detected in 77% of *Salmonella* isolates from chicken eggs [75]. Another study reported that the *aadA1* gene was detected in 97% of 135 *S*. Infantis isolates from Japan [16]. Acar et al. identified the *aadA1* gene in *S*. Infantis strains isolated from chicken meat [70]. The *aadA1* gene confers resistance by encoding the 3′-adenylate transferase enzymes, particularly streptomycin and spectinomycin. This gene is rapidly transferred between numerous bacterial species, mostly through class 1 integrons [74,76]. Although streptomycin has not been legally used in Türkiye in recent years, due to its long half-life and environmental persistence, this gene may be present in isolates [77]. The *aadA1* gene was not detected in the *S*. Infantis 1 isolate, which may be due to the presence of other *aadA* or *strA/B* gene variants that were not investigated in our study.

The *aphA1-IAB* gene inactivates aminoglycosides such as kanamycin and neomycin through phosphorylation [78]. The *aphA1-IAB* gene can be transferred via transposons such as Tn903 [79]. The long-term and widespread use of aminoglycosides, especially in veterinary medicine, has caused this gene to be under selective pressure in zoonotic bacteria such as *Salmonella* [69,80]. The *aphA1-IAB* gene was reported to be detected in 4.4% of 135 *S*. Infantis isolates in Japan [71]. In Türkiye, the presence of the *aphA1-IAB* gene has been shown in *S*. Infantis isolates isolated from chicken meat in Şanlıurfa, Bolu, and Ankara [71,81].

Our findings indicated resistance to trimethoprim and sulfamethoxazole in all three *S*. Infantis isolates using disk diffusion tests, and the sul1 gene was identified. The variant of the dihydropteroate synthase enzyme that confers resistance to sulfonamides is encoded by the *sul1* gene and is frequently associated with class 1 integrons [82]. The simultaneous identification of integron-associated genes such as *aadA1* and *sul1* introduces the possibility that these isolates may have developed resistance via mobile genetic elements. Furthermore, this process is known as the effect of co-selection because additional resistance genes on the same mobile genetic element can undergo co-selection under antibiotic pressure, such as trimethoprim and aminoglycosides [79]. This raises the possibility that zoonotic bacteria contain antibiotic resistance genes, particularly when combined with the widespread use of antibiotics for prophylaxis or treatment in animal production. The sul1 gene was detected in 70% of *S*. Indiana isolates isolated from frozen chicken meat in China [83]. It has been reported that the *sul1* gene is present in 77.78% of *S*. Enteritidis and *S*. Typhimurium isolates in Türkiye [84]. The identification of this gene, particularly in zoonotic pathogens, highlights the need to address antimicrobial resistance throughout the food distribution chain and in clinics [17].

Although tetracycline resistance was demonstrated in the antibiogram, the *tetA* and *tetB* genes were not found. This could be explained by the presence of more than 40 different *tet* resistance genes. In our study, genes encoding ribosomal protection proteins or mediating enzymatic inactivation were not detected because their primers were not included in the analysis.

### 4.3. Evolution of the Antimicrobial Effects of Carvacrol, Eugenol, and α-Terpineol on Salmonella Isolates

#### 4.3.1. Evolution of Minimum Inhibitory (MIC) and Bactericidal Concentration (MBC) Assay Values

One of the most effective preventive approaches against antibiotic-resistant food pathogens is to apply natural products with antibacterial activity to foods. Therefore, food-grade carvacrol, alpha-terpineol, and eugenol were used in our study due to their antibacterial activity. The MBC value of carvacrol for isolates S2 and S3 was 3.125 µL/mL. However, the MBC value of isolate S1 was 6.25. This difference suggests that carvacrol may be bactericidal at lower concentrations in isolates S2 and S3 and exhibits more potent antimicrobial activity against these strains. The results of this study are consistent with those reported by Dhifi et al., who reported that the most frequently used natural antibacterial agents, especially against Gram-negative bacteria, are phenolic compounds (thymol and carvacrol), aldehydes (cinnamaldehyde), and terpenes (eugenol and limonene) [85]. These active compounds disrupt the structural integrity of bacterial cell membranes, denature proteins, and negatively affect enzymatic activity, resulting in bacterial inhibition. Recent studies have shown that such bioactive compounds are effective in preserving the microbiological quality of foods [86]. The antibacterial effects of essential oil compounds, such as thymol, carvacrol, citral, and cinnamaldehyde, on *E. coli* O157:H7 and *S*. Typhimurium were investigated, and findings similar to ours were obtained [87]. These studies also reported that carvacrol-treated groups completely inhibited live cells within 60 and 30 min in the time-kill experiment, respectively. Khan et al. showed that carvacrol has a potent and rapid bactericidal effect against *E. coli* O157:H7 and *S*. Typhimurium pathogens in their studies [88]. Heckler et al. examined the antimicrobial effects of carvacrol and thymol against *Staphylococcus aureus* and *Salmonella* Enteritidis. They found the MIC value to be 200 µg/mL against both bacteria [89]. In another study conducted in Türkiye, the inhibitory effects of carvacrol, curcumin, and frankincense oil on *S*. Enteritidis and *S*. Enteritidis PT4 were investigated. The MIC values of carvacrol, curcumin, and frankincense oil were determined as 125.0 µg/mL, 132.5 µg/mL, and 31.3 mg/mL, respectively [90]. The MIC value of carvacrol against *Salmonella* Typhimurium was determined as 0.6 mg/mL, and the contact time required for bacterial inhibition was determined as 10 min [91]. In a similar study, the MICs and MBCs of carvacrol on *S.* Typhimurium were reported as 0.5 mg/mL and 1 mg/mL, respectively [92].

In our study, the MIC value of eugenol was determined to be within the range of 3.125–6.25 µL/mL, and the MBC value was 12.5 µL/mL for all isolates. It has been reported that the MIC values of eugenol against five clinical *S*. Typhimurium isolates varied between 0.00625% and 0.025% (*v*/*v*), and MBC values ranged between 0.0125% and 0.025% (*v*/*v*). In addition, the MIC values of 0.0125% and MBC values of 0.025% (*v*/*v*) were observed for the standard strain SL1344 [93,94]. The antimicrobial activity of eugenol was investigated with respect to three biofilm-forming isolates of ten *S*. Enteritidis isolates obtained from different chicken farms in Korea and the reference strain *S*. Enteritidis ATCC 13076. In this study, MIC values were reported to be 0.36% and 0.72% in the isolates and the reference strain, respectively [95]. As carried out by Balyan et al., Eugenol was encapsulated using an exopolysaccharide derived from *Lactobacillus plantarum* to increase its stability and antimicrobial activity. Their results showed that MBC values ranged from 2.6 to 4.4 mg/mL for *L. monocytogenes* and 1.6 to 3.2 mg/mL for *Salmonella*, with the lowest being 0.08 mg/mL for *E. coli*. They also found that MIC values varied depending on the strain [96].

When examining the MIC values of alpha-terpineol, the MIC value of isolates S1 and S2 was 6.25 µL/mL, while it was 12.5 µL/mL for isolate S3. The MBC value of all isolates was found to be 25 µL/mL. Similarly to our results, in a study evaluating the antimicrobial effects of α-terpineol, α-pinene, and 1,8-cineole on Gram-positive (*S. aureus* ATCC 25923, *L. monocytogenes* ATCC 1911, and *Bacillus cereus* ATCC 14579) and Gram-negative (*S*. Enterica ATCC 43972, and *E. coli* ATCC 25922) bacterial strains, it was reported that all three compounds exhibited significant antibacterial activity against five tested bacterial strains. In a study conducted by Gökmen, the antibacterial activities of carvacrol, α-terpineol, and eugenol were evaluated against extended-spectrum β-lactamase (ESBL)-producing *E. coli* strains isolated from chicken meat, and they were comparable to the scope of the present study. The broth dilution test revealed MIC and MBC values of 0.78 µL/mL for carvacrol, 3.125–6.25 µL/mL and 6.25–12.50 µL/mL for eugenol, and 3.125–6.25 µL/mL and 3.125–12.50 µL/mL for α-terpineol [97]. These results further support our findings by demonstrating that carvacrol exhibits a more potent antimicrobial effect at lower concentrations compared to the other tested compounds. The consistency between the two studies highlights the potent antimicrobial potential of carvacrol, particularly against multidrug-resistant Gram-negative pathogens, suggesting that it may play a promising role as a natural alternative in the control of foodborne bacteria.

In this study, carvacrol was identified as the most effective antimicrobial agent against *S*. Infantis isolates, while eugenol and α-terpineol exhibited relatively lower levels of activity. This discrepancy may be attributed to differences in the chemical structures, lipophilic properties, and modes of action of the tested compounds, in addition to isolate-specific resistance mechanisms. Carvacrol’s ability to induce membrane depolarization, disrupt bacterial cell integrity, and induce oxidative stress likely explains its effectiveness at lower concentrations. In contrast, the effects of eugenol and α-terpineol on membrane permeability appear to be more limited, which may explain their relatively weaker antimicrobial performance. This study also highlights the varying efficacy levels of natural antimicrobial agents against MDR *S*. Infantis strains and supports the potential of these three bioactive compounds (especially carvacrol) as alternative biological control agents. However, larger-scale studies are needed to comprehensively evaluate their efficacy against a wider range of bacterial strains.

#### 4.3.2. Evolution of Agar Well Diffusion Method Results

In this study, the strongest activity in the agar well diffusion test was observed in carvacrol, showing inhibition zones of 32–35 mm. A comparable investigation examining the antibacterial effect of carvacrol against *S*. Typhimurium found that the application of 0.25 and 0.5 mg carvacrol concentrations, respectively, resulted in mean inhibition zones of 13.1 mm and 18.3 mm, which is consistent with our findings [96]. The antibacterial activities of thymol and carvacrol against Gram-negative bacteria, including *Pseudomonas aeruginosa, E. coli*, and *S*. Typhimurium, and antifungal activities against *Candida albicans* were investigated by Akermi et al. The inhibition zones in the carvacrol-treated groups ranged from 18 to 23 mm, while those in the thymol-treated groups ranged from 14 to 21 mm, with carvacrol reported to have significantly stronger effects than thymol (*p* < 0.05) [98]. Additionally, the antimicrobial activity of eugenol against *E. coli* strains was evaluated using the disk diffusion method, and mean inhibition zone diameters of 38 ± 5 mm, 30 ± 4 mm, and 18 ± 4 mm were obtained at concentrations of 1000, 500, and 250 µg/mL, respectively [99]. Hussein et al. reported in vitro inhibition zones of α-terpineol against *S*. Typhimurium as 2.33, 2.09, and 1.99 mm at 24, 48, and 72 h, respectively. The in vivo application of α-terpineol was also observed to completely inhibit *Pseudomonas lundensis, Listeria monocytogenes*, and *S*. Typhimurium in ground chicken stored at 4 °C for two weeks [36]. Discs impregnated with eugenol at 1%, 5%, and 10% (*v*/*v*) concentrations on *S*. Typhi exhibited inhibition zones of 7 mm, 11 mm, and 11 mm, respectively, while discs containing ciprofloxacin (500 ng/mL), used as a positive control, exhibited an inhibition zone of 13 mm. These results indicate that eugenol exhibits significant antibacterial activity against *S*. Typhi, especially at high concentrations, and that its activity is similar to that of the conventional antibiotic ciprofloxacin [100]. The antimicrobial effects of various plant essential oils were evaluated, including clove oil produced in Türkiye by Evrendilek [101], which contains 67.3% eugenol, on common foodborne Gram-negative and Gram-positive pathogenic bacteria. It showed significant antimicrobial activity against various foodborne pathogens [100]. The antibacterial activities of carvacrol, α-terpineol, and eugenol were evaluated against ESBL-producing *E. coli* isolates by Gökmen [97]. In the eugenol-treated groups, inhibition zones were determined as 35 mm for isolates CM1 and CM3, while isolates CM2 and CM4 exhibited slightly smaller zones of 30 mm [97]. Jeyakumar et al. evaluated the antibacterial effect of eugenol on *E. coli* isolates using the well diffusion method and reported the inhibition zone diameter as 24 mm [102].

## 5. Conclusions

In conclusion, the findings of this study demonstrate that *S*. Infantis isolates exhibit multidrug resistance (MDR), mediated not only by plasmid-borne genes (*blaTEM-1*, *aadA1*, *aphA1-IAB*, and *sul1*) but also, potentially, by chromosomal mutations (*gyrA/parC* QRDR). The presence of such MDR patterns in animal-derived isolates poses significant concerns, as it restricts therapeutic options in veterinary medicine and represents a public health threat due to the risk of zoonotic transmission. Therefore, the continuous monitoring of isolates at both the phenotypic and genetic levels is essential in order to improve our understanding of resistance mechanisms and inform the development of effective control strategies.

This study also compared the antibacterial properties of carvacrol, α-terpineol, and eugenol against *Salmonella* Infantis isolates from commercially marketed chicken meat produced in various integrated facilities. The findings revealed that all three compounds exhibited significant antimicrobial activity (*p* < 0.05). The compound with the strongest antibacterial activity was carvacrol, which had low MIC/MBC values and large inhibition zone diameters. In vitro, α-terpineol showed promising results. Eugenol exhibited antibacterial activity similar to antibiotics at high concentrations, although it was less effective than carvacrol and α-terpineol with respect to certain strains.

## Figures and Tables

**Table 1:** Primers For Antibiotic Resistance Gene.

Gene	Sequences	°C *	bp	References
*TEM*	F: CATTTCCGTGTCGCCCTTATTC R: CGTTCATCCATAGTTGCCTGAC	52 °C	800 bp	[41,42,43]
*SHV*	F: AGCCGCTTGAGCAAATTAAAC R: ATCCCGCAGATAAATCACCAC	52 °C	713 bp	
*OXA*	F: GGCACCAGATTCAACTTTCAAG R: GACCCCAAGTTTCCTGTAAGTG	52 °C	564 bp	
*CTXM1*	F: TTAGGAARTGTGCCGCTGYA R: CGATATCGTTGGTGGTRCCAT	52 °C	688 bp	
*CTXM2*	F: CGTTAACGGCACGATGAC R: CGATATCGTTGGTGGTRCCAT	52 °C	404 bp	
*CTXM9*	F: TCAAGCCTGCCGATCTGGT R: TGATTCTCGCCGCTGAAG	52 °C	561 bp	
*CTXM* *8/25*	F: AACRCRCAGACGCTCTAC R: TCGAGCCGGAASGTGTYAT	52 °C	326 bp	
*TEM-1*	F: CAGCGGTAAGATCCTTGAGA R: ACTCCCCGTCGTGTAGATAA	46 °C	643 bp	[44]
*tetA*	F: GGTTCACTCGAACGACGTCA R: CTGTCCGACAAGTTGCATGA	55 °C	210 bp	[41]
*tetB*	F: CCTCAGCTTCTCAACGCGTG R: GCACCTTGCTGATGACTCTT	55 °C	659 bp	
*cat1*	F: ATGAGAAAAAATCACTGGATATACC R: TTACGCCCCGCCCTGCC	56 °C	547 bp	[44,45]
*cat2*	F: TCCGGGCCTGCTGACAGGCATC R: GAGTTGAGCGTCAGCGGGTG	56 °C	352 bp	
*qnrA*	F:GGATGCCAGTTTCGAGGA R:TGCCAGGCACAGATCTTG	50 °C	492 bp	[46,47,48]
*qnrB*	F:GGMATHGAAATTCGCCACTG R:TTTGCYGYYCGCCAGTCGAA	50 °C	264 bp	
*qnrS*	F:TCGACGTGCTAACTTGCG R:GATCTAAACCGTCGAGTTCGG	50 °C	466 bp	
*qnrC*	F:GGGTTGTACATTTATTGAATCG R:CACCTACCCATTTATTTTCA	50 °C	307 bp	
*qnrD*	F:CGAGATCAATTTACGGGGAATA R:AACAAGCTGAAGCGCCTG	50 °C	582 bp	
*aadA1*	F:TATCAGAGGTAGTTGGCGTCAT R:GTTCCATAGCGTTAAGGTTTCATT	45 °C	484 bp	[41]
*aphA1-IAB*	F: AAACGTCTTGCTCGAGGC R: CAAACCGTTATTCATTCGTGA	46 °C	500 bp	
*Sul1*	F: TCACCGAGGACTCCTTCTTC R: CAGTCCGCCTCAGCAATATC	45 °C	331 bp	[49]
*ermB*	F:GAAAAGGTACTCAACCAAATA R:AGTAACGGTACTTAAATTGTTTAC	52 °C	639 bp	[50]

* °C: Annealing temperature.

**Table 2:** Distribution of *Salmonella* spp. according to chicken meat sample.

Sample	*Salmonella* spp (n, %)	*S*. Typhimurium (n, %)	*S*. Infantis (n, %)	*S*. Enteritidis (n, %)
Wing (n: 25)	ND	ND	ND	ND
Breasts (n: 25)	ND	ND	ND	ND
Drumstick (n: 25)	1, 4%	ND	%4	ND
Thigh (n: 25)	2, 8%	ND	%8	ND

**Table 3:** Antibiotic susceptibility of *Salmonella* Infantis isolates determined by the disk diffusion method.

Antibiotics	*Salmonella* Infantis 1 (S1)	*Salmonella* Infantis 2 (S2)	*Salmonella* Infantis 3 (S3)
Ampicillin-AM	R	R	R
Amoxicillin-clavulanic acid-AMC	R	R	R
Ceftriaxone-CRO	R *	R	S
Ceftazidime-CAZ	S	S	S
Aztreonam-ATM	S	S	S
Cefotaxime-CTX	S	S	S
Gentamicin-CN	S	S	S
Amikacin-AK	S	S	S
Ciprofloxacin-CIP	S	S	S
Levofloxacin-LEV	S	S	S
Enrofloxacin-ENR	S	S	S
Trimethoprim-sulfamethoxazole-TMZ	R	R	R
Tetracycline-T	R	R	R
Chloramphenicol-C	S	S	S
Streptomycin-S	R	R	R
Kanamycin-K	R	R	R

*: Suspicious borderline zone, R: Resistant, S: Sensitive.

**Table 4:** The Minimum Inhibitory Concentration (MIC) and Minimum Bactericidal Concentration (MBC) values of bioactive compounds.

*Salmonella* Infantis Isolate	Minimum Inhibitory Concentration (MIC)			Minimum Bactericidal Concentration (MBC)		
	Carvacrol	Eugenol	α-Terpineol	Carvacrol	Eugenol	α-Terpineol
S1	1.56 µL/mL	3.125 µL/mL	6.25 µL/mL	6.25 µL/mL	12.5 µL/mL	25 µL/mL
S2	1.56 µL/mL	6.25 µL/mL	6.25 µL/mL	3.125 µL/mL	12.5 µL/mL	25 µL/mL
S3	1.56 µL/mL	6.25 µL/mL	12.50 µL/mL	3.125 µL/mL	12.5 µL/mL	25 µL/mL

## Data Availability

The data sets used are available from the corresponding author upon reasonable request.
